# Anomalous Thermopower and High *ZT* in GeMnTe_2_ Driven by Spin's Thermodynamic Entropy

**DOI:** 10.34133/2021/1949070

**Published:** 2021-03-11

**Authors:** Sichen Duan, Yinong Yin, Guo-Qiang Liu, Na Man, Jianfeng Cai, Xiaojian Tan, Kai Guo, Xinxin Yang, Jun Jiang

**Affiliations:** ^1^Ningbo Institute of Materials Technology and Engineering, Chinese Academy of Science, Ningbo 315201, China; ^2^School of Materials Science and Engineering, Shanghai University, Shanghai 200444, China; ^3^Center of Materials Science and Optoelectronics Engineering University of Chinese Academy of Sciences, Beijing 100049, China

## Abstract

Na_*x*_CoO_2_ was known 20 years ago as a unique example in which spin entropy dominates the thermoelectric behavior. Hitherto, however, little has been learned about how to manipulate the spin degree of freedom in thermoelectrics. Here, we report the enhanced thermoelectric performance of GeMnTe_2_ by controlling the spin's thermodynamic entropy. The anomalously large thermopower of GeMnTe_2_ is demonstrated to originate from the disordering of spin orientation under finite temperature. Based on the careful analysis of Heisenberg model, it is indicated that the spin-system entropy can be tuned by modifying the hybridization between Te-*p* and Mn-*d* orbitals. As a consequent strategy, Se doping enlarges the thermopower effectively, while neither carrier concentration nor band gap is affected. The measurement of magnetic susceptibility provides a solid evidence for the inherent relationship between the spin's thermodynamic entropy and thermopower. By further introducing Bi doing, the maximum *ZT* in Ge_0.94_Bi_0.06_MnTe_1.94_Se_0.06_ reaches 1.4 at 840 K, which is 45% higher than the previous report of Bi-doped GeMnTe_2_. This work reveals the high thermoelectric performance of GeMnTe_2_ and also provides an insightful understanding of the spin degree of freedom in thermoelectrics.

## 1. Introduction

Thermoelectrics offers a unique solution for the direct conversion from waste heat into versatile electricity [1–3]. The commercial applications of thermoelectric device, however, are still limited by the low conversion efficiency. The performance of thermoelectrics is evaluated by the dimensionless figure of merit, *ZT* = *α*^2^*σT*/*κ*_*e*_ + *κ*_*ph*_, where *α*, *σ*, *κ*_*e*_, *κ*_*ph*_, and *T* are the Seebeck coefficient, electrical conductivity, electrical thermal conductivity, lattice thermal conductivity, and absolute temperature, respectively [4–14]. Since the transport factors are strongly coupled with each other, to achieve a high *ZT* is always a big challenge.

In principle, a physical phenomenon in a solid can be understood on the basis of the four fundamental degrees of freedom, i.e., charge, lattice, spin, and orbital [14–18]. Thermoelectric transport involves the charge and lattice degrees of freedom obviously. The most applied strategies for performance enhancement can be attributed to the handling of the charge and lattice degrees of freedom. On the other hand, the effects of orbital and spin degrees of freedom in thermoelectrics are not that evident. Until recent years, the crucial role of orbital degree freedom in the scheme of band engineering was gradually disclosed by theoretical studies [19–21]. In many systems, such as PbTe, SnTe, and Mg_2_Si, band engineering has been proved to be a very efficient strategy for improving *ZT*, since it partly decouples the electrical conductivity and Seebeck coefficient [21–27]. The examples for spin degree of freedom in thermoelectrics are rare. Na_*x*_CoO_2_ exhibits an anomalous Seebeck coefficient [28, 29], which is nearly one order higher than the prediction from Mott's formula [30]. This puzzle was finally resolved by the concept of spin entropy [29, 31]. Owing to the increased spin degeneracy, the large entropy current strongly enhances the Seebeck coefficient in Na*_x_*CoO_2_. It is conceivable that a system with more adjustable degrees of freedom has the larger potential to be improved. “*Thermoelectrics needs a spin*,” it was addressed by J. He and T. Tritt recently [14].

Despite the significantly enhanced Seebeck coefficient, Na*_x_*CoO_2_ has a moderate maximum *ZT* around 1 at 800 K [32], whereas the maximum *ZT* in some traditional semiconductors, such as PbTe, GeTe, and SnSe, has been improved above 2 [33–35]. The limited *ZT* in Na_*x*_CoO_2_ may be owing to two reasons. First, the localized *d*-orbital of Co strongly reduces the carrier mobility, although it is also the origin of spin entropy. Second, the microscopic understanding of spin entropy is not insightful enough to make the further optimization. The original spin entropy explanation for Na_*x*_CoO_2_ is an approximation with high-temperature limit (*T* → +∞), i.e., the spin entropy takes its maximum, and the deduced Seebeck coefficient is temperature-independent [29]. For this reason, the theory does not tell us how to tune the spin entropy under finite temperature. Recently, the Seebeck coefficient in Li-doped MnTe was found to be significantly enhanced by the magnetic phase transition, while the enhancement is strongly dependent on the transition temperature and carrier concentration [36–38]. The phenomenon was explained by magnon (or paramagnon) drag, but not spin entropy. Nevertheless, this study gives some clues about the optimization of the spin degree of freedom. The maximum *ZT* of Li-doped MnTe reaches 1 at 900 K [36].

The IV-VI compound GeTe is a remarkable thermoelectric material characterized by high electrical conductivity and high power factor *PF* = *α*^2^*σ* [34, 39–45]. GeTe undergoes a phase transition from rhombohedral structure to cubic structure at 700 K [46]. When 50% Ge is substituted by Mn, the cubic phase is stabilized in GeMnTe_2_ at room temperature [47, 48]. GeMnTe_2_ was found to have a very small band gap less than 0.05 eV, while its Seebeck coefficient reaches 200 *μ*V/K at 700-800 K. According to the Goldsmid-Sharp relationship, *E*_*g*_ = 2*eS*_max_*T*(*S*_max_) [49], the estimated band gap is around 0.3 eV, that is to say, the measured Seebeck coefficient is nearly one order higher than the theoretical prediction. The presence of the 3*d* transition element Mn in GeMnTe_2_ induces a ferromagnetic phase transition below room-temperature [50]. Comparing with the examples of Na*_x_*CoO_2_ and MnTe, it is reasonable to presume that the enhancement of Seebeck coefficient is related to the spin degree of freedom. Previous studies on GeMnTe_2_ have treated the system as a traditional semiconductor. Bi doping and Pb doping have been applied to optimize the performance, while the peak *ZT* of 0.9 and 1.4 were achieved, respectively [47, 48]. To study the spin degree of freedom in GeMnTe_2_ is not only theoretically meaningful, and it may also promote the realistic application of this material.

In this work, the thermoelectric properties of GeMnTe_2_ are investigated by manipulating the spin degree of freedom. Based on the analysis of Heisenberg model, two specific strategies are designed to enhance the spin's thermodynamic entropy. Se doping and Bi doping are introduced individually to reduce the spin-spin interaction by fully filling the *p*-orbital of anions. The Seebeck coefficient is found to be effectively enhanced, while the measurements of magnetic susceptibility prove that the enhancements are inherently related to the variation of entropy. In Ge_0.94_Bi_0.06_Te_1.94_Se_0.06_, a high peak *ZT* of 1.4 at 843 K is achieved. Compared with the previous report on singly Bi-doping, the maximum ZT is increased by 45%. This work shows GeMnTe_2_ is a promising thermoelectric material and also provides an insightful understanding on the spin degree of freedom in thermoelectrics.

## 2. Theoretical Analysis and Experiment Design

Seebeck coefficient and entropy are related via the electron chemical potential. The chemical potential of electron *μ* is given by *μ* = −*T*(*∂S*/*∂N*)_*U*,*V*_, where entropy *S* = *S*(*U*, *V*, *N*) is a function of energy *U*, volume *V*, and electron number *N*. Then, Seebeck coefficient *α* can be written as

(1) $$\begin{array}{c} \alpha =\frac{1}{e}\frac{\partial \mu }{\partial T}=-\frac{1}{e}\frac{\partial }{\partial T}\left(T\frac{\partial S}{\partial N}\right). \end{array}$$

Within high temperature limit, entropy is temperature independent, and equation becomes Heikes formula *α* = −(1/*e*)(*∂S*/*∂N*). The entropy *S* = *S*(*U*, *V*, *N*) includes the contributions from both charge and spin degrees of freedom. If spin degree of freedom is frozen, its contribution to entropy is zero, and the given Seebeck coefficient should obey the Goldsmid-Sharp relationship [49]. In GeMnTe_2_, the estimated band gap from Goldsmid-Sharp relationship is one order higher than the measured one. This indicates that the spin degree of freedom dominates the Seebeck coefficient. Thus, the Seebeck coefficient could be further enhanced by simply increasing the spin entropy.

**Figure 1:**
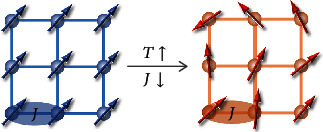
Schematic representation of spin orientation and thermodynamic entropy. As temperature *T* increases or exchange integral *J* decreases, the perturbation of spin order leads to the increase of entropy.

In Na*_x_*CoO_2_, the large entropy is from the high degeneracy of *d*-electron local states, owing to the competition between crystal field splitting and exchange splitting. It was argued that the coexistence of Co^3+^ and Co^4+^ ions plays an important role [29]. In GeMnTe_2_, the 3*d* element Mn only has the +2 valence state, and therefore, the degeneracy of local states should be much lower than that in Na*_x_*CoO_2_. Another kind of spin entropy may be related to the spin orientation. As shown in Figure 1, the highly ordered spin orientation indicates a low entropy state. As temperature increases, the long-range order of spins is broken and the entropy increases. To calculate the energy-dependent entropy *S* = *S*(*U*, *V*, *N*) is difficult, especially for a spin-correlated system. But we still can get some useful information from qualitative analysis. The interaction of a spin system can be described by Heisenberg model:

(2) $$\begin{array}{c} H=-\underset{i,j}{\Sigma^{\prime }}J_{ij}{\hat{S}}_{i}\cdot {\hat{S}}_{j}, \end{array}$$

where *J* is the exchange integral

(3) $$\begin{array}{c} J_{ij}=\int \phi_{i}^{*}\left(r_{i}\right)\phi_{j}^{*}\left(r_{j}\right)V\left(r_{ij}\right)\phi_{i}\left(r_{j}\right)\phi_{j}\left(r_{i}\right)dr_{i}r_{j}. \end{array}$$

It is easy to find that the transition temperature of a spin-ordered state is determined by the magnitude of *J*. As for a given system, a larger ∣*J*∣ will lead to a higher transition temperature. The ordering of spin, i.e., entropy, depends on the competition between temperature *T* and exchange integral *J* (as shown in Figure 1). Under a certain temperature, the entropy from the spin degree of freedom could be enhanced by decreasing *J*. In Na*_x_*CoO_2_, “spin entropy” was referred to the highly degenerate spin states under high-temperature limit. Here, the entropy from the disordering of spin orientation under finite temperature is termed as spin's thermodynamic entropy, to distinguish it from the concept of spin entropy in Na*_x_*CoO_2_.

Equation (3) shows that the magnitude of *J*_*ij*_ depends on the overlap of *d*-wave functions at the spin sites of *i* and *j*. In GeMnTe_2_, the 3*d* elements are separated by Te^2-^ ions. Thus, the effective *d*-*d* hopping should be via Te-*p* orbitals. The stronger *p*-*d* hopping is, the larger *J* is. If Te-*p* orbitals are fully filled by the Mn-*s* electrons, the *p*-*d* hopping will be screened, leading to a zero *J*. In the real system, *p*-*d* hopping cannot be completely screened because the energy levels of Mn-*s* and Mn-*d* are close. According to above discussions, two strategies can be figured out to reduce *J*. The first is to replace Te with the elements having stronger electronegativity. The second is to reduce the carrier concentration. The both two strategies can reduce the *p*-*d* hopping by further filling the Te-*p* orbitals. In this work, Se doping is designed to increase the electronegativity of anions, and Bi doping is designed to reduce the carrier concentration.

## 3. Results and Discussion

**Figure 2:**
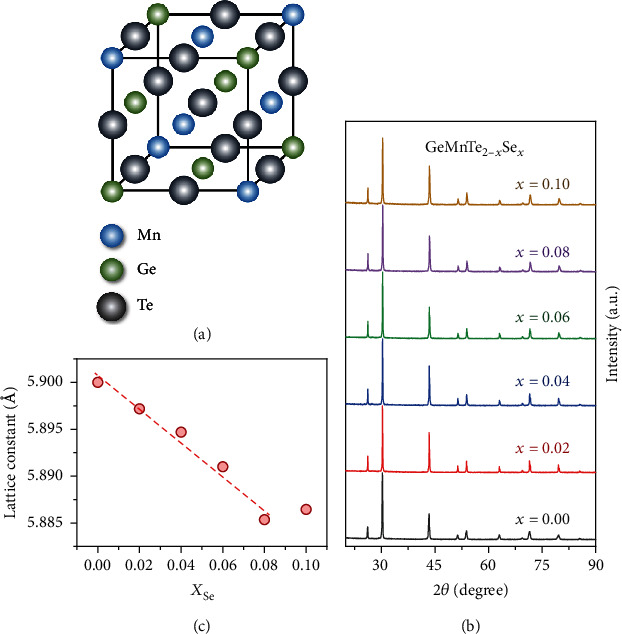
Measured crystal structure of GeMnTe_2-__*x*_Se_*x*_. (a) Crystal structure for cubic GeMnTe_2_, (b) powder XRD patterns, and (c) calculated lattice constants for GeMnTe_2-__*x*_Se_*x*_ (*x* = 0, 0.02, 0.04, 0.06, 0.08, 0.10).

GeMnTe_2_ has a NaCl type structure, with Ge and Mn occupying the Na sites equally (as shown in Figure 2(a)). Figure 2(b) presents the XRD patterns for the examples with Se doping. All the samples are indicated to be the rock-salt structure, and no secondary phases can be observed from the XRD patterns. Figure 2(c) displays the calculated lattice constants from XRD results. The obtained lattice constants of GeMnTe_2-__*x*_Se_*x*_ show a linear decrease as the amount of Se increases up to *x* = 0.08. At *x* = 0.10, the XRD patterm still indicates a cubic structure, but the lattice constant does not obey the linear decrease. The measurements show that the GeMnTe_2-__*x*_Se_*x*_ (*x* = 0 − 0.08) samples are homogenous solid solutions.

**Figure 3:**
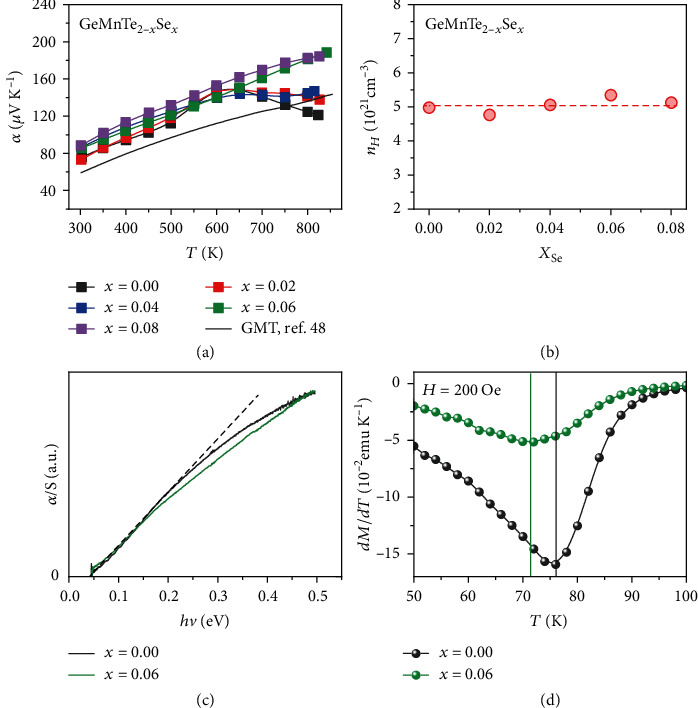
Measured electrical properties of GeMnTe_2-__*x*_Se_*x*_. (a) Temperature-dependent Seebeck coefficient of GeMnTe_2-__*x*_Se_*x*_ (*x* = 0 − 0.08). (b) Hall carrier concentration at room temperature as a function of Se content in GeMnTe_2-__*x*_Se_*x*_ (*x* = 0 − 0.08). (c) Optical band gap from infrared absorption spectra and (d) the temperature-dependent magnetization derivative *dM*/*dT* for GeMnTe_2-__*x*_Se_*x*_ (*x* = 0, 0.06).

The measured Seebeck coefficients for GeMnTe_2-__*x*_Se_*x*_ are displayed in Figure 3(a) as a function of temperature. It may be seen that Se doping increases the Seebeck coefficient within a large temperature range. At 840 K, GeMnTe_1.94_Se_0.06_ shows a high Seebeck coefficient about 190 *μ*V/K, compared the value of 120 *μ*V/K for the undoped GeMnTe_2_. The thermoelectric properties of GeMnTe_2_ has been reported by Zhou et al., and they used similar synthesis method [48]. Compared with their data, our Se-doped samples also show the largely enhanced Seebeck coefficients. The measured electrical conductivity and power factor are presented in the Supplementary Information (available here). The sample of GeMnTe_1.94_Se_0.06_ is found to have the largest power factor of 15 *μ*wcm^−1^ K^−2^ at 840 K.

Figure 3(b) presents the Hall carrier concentrations of GeMnTe_2-__*x*_Se_*x*_ at room temperature. The measured carrier concentrations are all around 5 × 10^21^ cm^−3^. It is not surprising that the substitution of Se for Te does not change the carrier concentration. Figure 3(c) shows the optical band gap from infrared absorption spectra. The both two samples of *x* = 0 and *x* = 0.06 show the very small band gap less than 0.05 eV. This result is consistent with the previous report [47]. We applied band structure calculations for GeMnTe_2_, but failed to produce a band gap. It is not uncommon that density functional theory fails to describe the electron-correlated system. Then, the enhancement of Seebeck coefficient in Se-doped samples is neither from the variation of band gap nor from the decreasing carrier concentration.

As we discussed, Se doping was designed to increase the spin's thermodynamic entropy by reducing the exchange integral *J*. GeMnTe_2_ was found to have a ferromagnetic phase transition below room temperature. If *J* is reduced by Se doping, the magnetic transition temperature *T*_*C*_ should decrease. To verify this purposive design, we measured the low temperature magnetic susceptibility for *x* = 0 and *x* = 0.06 samples. Using the measured magnetic susceptibility, the derivative of magnetization *dM/dT* can be calculated. The Curie temperature *T*_*C*_ can be roughly determined by the minimum of *dM/dT*. In Figure 3(d), the deduced *dM/dT* is plotted as a function of temperature. As may be seen, the estimated transition temperature *T*_*C*_ is decreased from 76 K for *x* = 0 to 71 K for *x* = 0.06. The enhanced Seebeck coefficient and decreased Curie temperature by Se doping are consistent with our experiment design, indicating the crucial role of spin's thermodynamic entropy in GeMnTe_2_.

In the study of Zhou et al., GeMnTe_2_ was treated as a traditional semiconductor, and Bi doping was introduced to adjust the carrier concentration [48]. At room temperature, the Seebeck coefficient was increased from 55 *μ*V/K for GeMnTe_2_ to 140 *μ*V/K for Ge_0.9_Bi_0.1_MnTe_2_, while the hole concentration is decreased from 3.6 × 10^21^ cm^−3^ to 1.0 × 10^21^ cm^−3^. Considering the high level of carrier concentration, the measured Seebeck coefficient is unusually large. For comparison, the Seebeck coefficient of GeTe is about 100 *μ*V/K at room temperature with the carrier concentration of 1.5 × 10^20^ cm^−3^ [34]. The large enhancement of Seebeck coefficient by Bi doping may not be simply explained by the decrease of carrier concentration. As we discussed, the decrease of carrier concentration could increase the spin's thermodynamic entropy, which should be the underlying reason for the increase Seebeck coefficient. To clarify this problem, Bi doping is introduced into the GeMnTe_1.94_Se_0.06_ sample.

**Figure 4:**
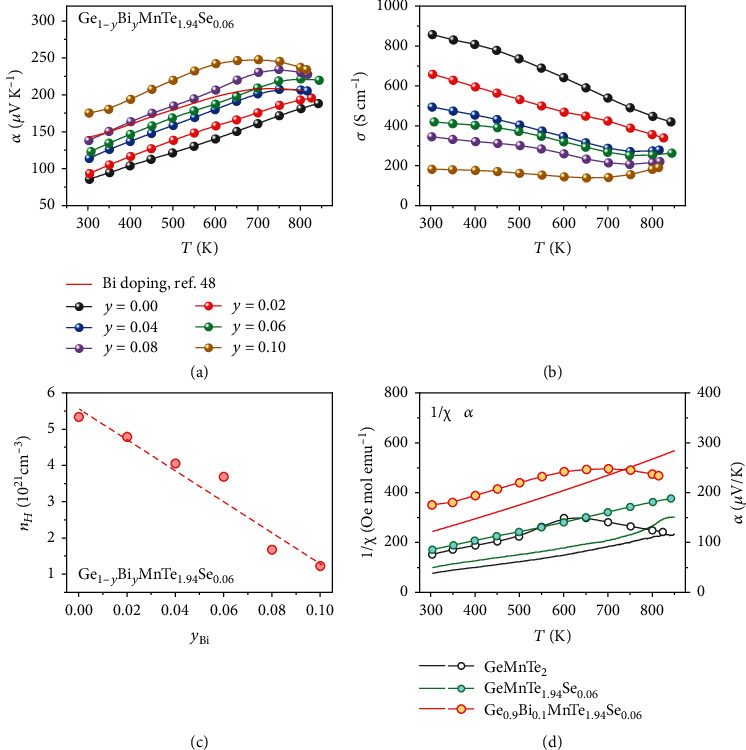
The Thermoelectric properties of Ge_1-__*y*_MnBi_*y*_Te_1.94_Se_0.06_. Temperature-dependent Seebeck coefficients (a) and electrical conductivity (b) for Ge_1-__*y*_Bi_*y*_MnTe_1.94_Se_0.06_, and Hall carrier concentration (c) at room temperature as a function of Bi doping level, (*y* = 0, 0.02, 0.04, 0.06, 0.08, 0.10). The inherently relationship between magnetic susceptibility and Seebeck coefficient is displayed in (d).

Figure 4(a) shows the measured Seebeck coefficient for Ge_1-__*y*_Bi_*y*_MnTe_1.94_Se_0.06_. It may be seen that the Seebeck coefficient shows a monotonous increase as Bi doping increases from *y* = 0 to *y* = 0.1. At room temperature, Seebeck coefficient is significantly enhanced from 85 *μ*V/K for *y* = 0 to 175 *μ*V/K for *y* = 0.1. In Figure 4(a), the data of Ge_0.9_Bi_0.1_MnTe_2_ from Zhou et al. are also presented [48]. Compared with the singly Bi-doped sample, the Bi/Se codoped sample shows the higher Seebeck coefficient. Figure 4(b) show the electrical conductivity for all the samples. As the amount of Bi doping increases, electrical conductivity is decreased monotonously. In Figure 4(c), the room-temperature Hall carrier concentrations of Ge_1-__*y*_Bi_*y*_MnTe_1.94_Se_0.06_ are presented. The measured carrier concentration continuously decreases from 5.3 × 10^21^ cm^−3^ for *y* = 0 to 1.2 × 10^21^ cm^−3^ for *y* = 0.1. The carrier concentration of Ge_0.9_Bi_0.1_MnTe_1.94_Se_0.06_ is very closed to the reported value of Ge_0.9_Bi_0.1_MnTe_2_ [48]. This is consistent with our conclusion that Se doping does not change the carrier concentration.

According to our discussions, Bi doping could increase the spin's thermodynamic entropy by reducing the carrier concentration. It is difficult to measure the entropy directly. The ordering of spin orientation, however, can be characterized by the magnetic susceptibility under weak field. GeMnTe_2_ has a ferromagnetic phase at low temperature [48]. If the spin orientation is well ordered, the system should have a high magnetic susceptibility and a low entropy. Figure 4(d) displays the measured magnetic susceptibility for 3 representative samples, GeMnTe_2_, GeMnTe_1.94_Se_0.06_, and Ge_0.9_Bi_0.1_MnTe_1.94_Se_0.06_. The reciprocals of magnetic susceptibility for all the samples show the good linear dependence on temperature within a large temperature range. Figure 4(d) clearly shows that magnetic susceptibility are strongly correlated to Seebeck coefficient. GeMnTe_2_ has the largest magnetic susceptibility and the smallest Seebeck coefficient, GeMnTe_1.94_Se_0.06_ has the intermediate magnetic susceptibility and the intermediate Seebeck coefficient, while Ge_0.9_Bi_0.1_MnTe_1.94_Se_0.06_ has the smallest magnetic susceptibility and the largest Seebeck coefficient. Furthermore, the differences between magnetic susceptibility is found to be in proportion to the difference between Seebeck coefficient. It is noticeable that the variation of Seebeck coefficient between the three samples cannot be explained by Mott's expressions. The Goldsmid-sharp relationship gives the limit of the maximum of Seebeck coefficient. As we discussed, the measured small band gap indicates that the Goldsmid-sharp relationship fails to describe the thermopower of GeMnTe_2_. Figure 4(d) presents a convincing evidence for the presumption of spin's thermodynamic entropy.

**Figure 5:**
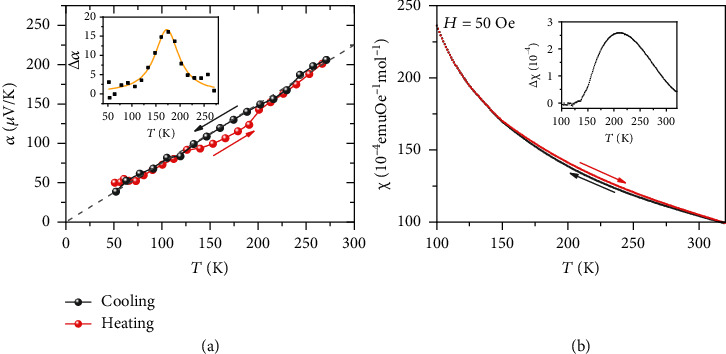
Low temperature Seebeck coefficient and magnetic susceptibility of Ge_0.9_Bi_0.1_MnTe_1.94_Se_0.06_ measured with two processes of heating and cooling. (a) Low temperature Seebeck coefficient and (b) low temperature magnetic susceptibility. The insets in (a) and (b) are the difference of Seebeck coefficient and magnetic susceptibility between the cooling and heating progress, respectively.

Figure 5(a) presents the low temperature Seebeck coefficient from 50 K to 300 K. The Seebeck coefficient was measured in the two processes of cooling and heating. Interestingly, the measured Seebeck coefficients from two processes do not converge. From 120 K to 200 K, the cooling process gives higher Seebeck coefficient than the heating process does. In the traditional semiconductors, such phenomenon would be difficult to understand. In magnetic materials, however, the effect of magnetic hysteresis is common: magnetic systems tend to retain its spin alignment. In this measurement of Seebeck coefficient, no magnetic field was applied, but the spontaneous magnetization occurs at low temperature. In the heating process, the system stars at a low entropy state. At a certain temperature, the heating process may cause lower entropy than the cooling process, for the spin state is history-dependent. As shown in the inserted figure, the largest difference of Seebeck coefficient reaches 15 *μ*V/K at 170 K. Figure 5(b) shows the magnetic susceptibility under a low magnetic field of 50 Oe for the two processes of cooling and heating. As we expected, the heating process exhibits the lower magnetic susceptibility than the cooling process from 130 K to 300 K. Within the picture of spin's thermodynamic entropy, the measured Seebeck coefficient and magnetic susceptibility within two processes are consistent with each other. The history-dependent Seebeck coefficient confirms the strong effect from the spin degree of freedom.

**Figure 6:**
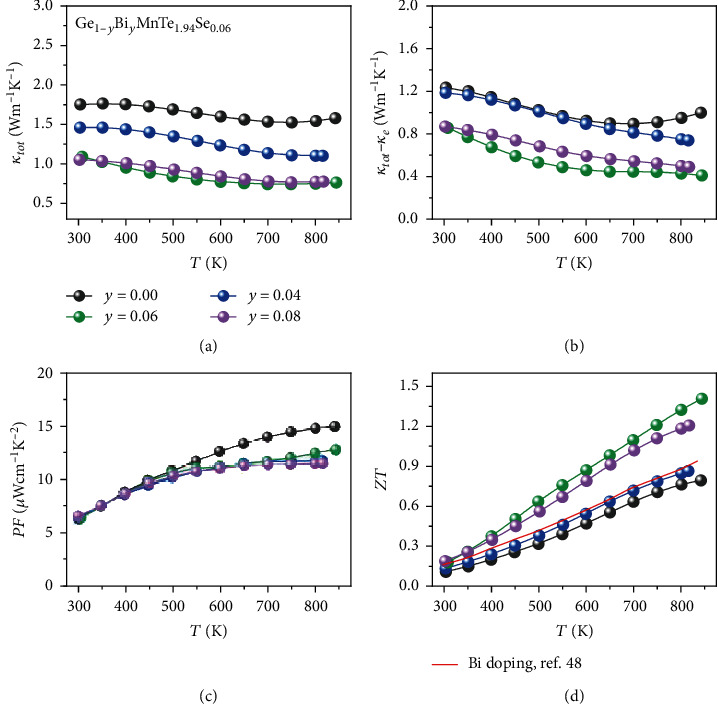
The thermoelectric performance of Ge_1-__*y*_MnBi_*y*_Te_1.94_Se_0.06_ (*y* = 0.04, 0.06, 0.08). Temperature-dependent total thermal conductivity (a), lattice thermal conductivity (b), power factor (c), and figure of merit *ZT* (d). The red solid line in (d) denotes the previous report on Bi-doped GeMnTe_2_ [48].

Figures 6(a) and 6(b) present the total thermal conductivity and lattice thermal conductivity for Ge_1-__*y*_Bi_*y*_MnTe_1.94_Se_0.06_, respectively. The lattice thermal conductivity is determined by relationship of *κ*_*L*_ = *κ*_*tot*_ − *κ*_*e*_, where *κ*_*tot*_ is the total thermal conductivity and *κ*_*e*_ is the electrical thermal conductivity. The Wiedemann-Franz relation *κ*_*e*_ = *LσT* is used to calculate the electrical thermal conductivity, where *L*, *σ*, and *T* are the Lorenz number, the electrical conductivity, and the absolute temperature, respectively. The Lorenz number is calculated based on the SPB model. As may be seen, the total thermal conductivity decreases as Bi doping increased up to *y* = 0.06. At *y* = 0.08, the total thermal conductivity is close to the one of *y* = 0.06. The lattice thermal conductivity of Ge_1-__*y*_Bi_*y*_MnTe_1.94_Se_0.06_ shows similar tendency to the total thermal conductivity. At *y* = 0.06, the lattice thermal conductivity reaches a minimum 0.41 at 840 K. At *y* = 0.08, the lattice thermal conductivity shows an increase relative to *y* = 0.06. Such nonmonotonic thermal conductivity in solid solutions is not uncommon.

Figure 6(c) shows the temperature-dependent power factors of Ge_1-__*y*_Bi_*y*_MnTe_1.94_Se_0.06_. The maximum *PF* appears at the *y* = 0 sample. With Bi doping, the peak of power factor is reduced from 15 *μ*Wcm^−1^ K^−2^ at *y* = 0.0 to 10 *μ*Wcm^−1^ K^−2^ at *y* = 0.10. The reduction of *PF* by Bi doping is mainly due to the decrease of electrical conductivity (as shown in Figure 4(b)). The obtained thermoelectric figure of merit for Ge_1-__*y*_Bi_*y*_MnTe_1.94_Se_0.06_ is displayed in Figure 6(d). Ge_0.94_Bi_0.06_MnTe_1.94_Se_0.06_ shows the maximum *ZT* of 1.4 at 840 K. Compared with the previous report on Bi-doped GeMnTe_2_, the maximum *ZT* is enhanced by 45%. The results show that the thermoelectric performance of is GeMnTe_2_ efficiently enhanced by Se doping. Therefore, our designed strategies are proved to be effective, and the picture of spin's thermodynamic entropy is verified.

Here, we show two effective strategies for the manipulation of the spin's thermodynamic entropy. According to our theoretical analysis, other effective strategies may also be expected. For example, if the crystal lattice can be enlarged, the exchange integral *J* will be reduced and the system band gap will be enlarged. This situation will lead to the cooperation of the charge and spin degrees of freedom. Therefore, the thermoelectric performance of GeMnTe_2_ is believed to have a large potential to be further improved.

## 4. Conclusion

In summary, the thermoelectric properties of the emerging materials GeMnTe_2_ are investigated. GeMnTe_2_ was found to have a large thermopower and a small band gap, which is apparently against the Goldsmid-Sharp relationship. By measuring the magnetic susceptibility, we demonstrate that the anomalous thermopower of GeMnTe_2_ is inherently related with the spin's thermodynamic entropy. Heisenberg model is implemented to analyse the entropy of the spin system. It is found that the entropy depends on the competition between temperature and exchange integral. Under finite temperature, the entropy can be enhanced by reducing the exchange integral, which could be achieved by decreasing the hybridization between Te-*p* and Mn-*d* orbitals. The consequent strategies, Se-doping and Bi-doping, are proved to be very efficient. In Ge_0.94_Bi_0.06_MnTe_1.94_Se_0.06_, the maximum *ZT* reaches 1.4 at 840 K, which is 45% higher than previous report. This work reveals a new system with high thermoelectric performance and provides an insight into manipulation of spin degree freedom in thermoelectrics.

## 5. Experimental Section

### 5.1. Synthesis

The compounds with nominal compositions of GeMnTe_1-__*x*_Se_*x*_ (*x* = 0 − 0.10) and MnGe_1-__*y*_Bi_*y*_Te_1.94_Se_0.06_ (*y* = 0 − 0.10) were prepared by vacuum melting combined with HP process. High purity bulk germanium (Ge, 99.999%), manganese (Mn, 99.999%), bismuth (Bi, 99.999%), tellurium (Te, 99.999%), and selenium (Se, 99.999%) were weighted according to the stoichiometric proportions to obtain the compounds. The raw materials were sealed into a vacuum quartz tube, melted and swung at 1223 K for 1 h, quenched in the cold water, and then annealed at 950 K for 72 h. The obtained ingots were crushed and hand ground into fine powders. The powders were vacuum hot pressed in a *ϕ*12.7 mm graphite mold at 900 K for 30 min under the uniaxial stress of 50 MPa to obtained dense bulk samples.

### 5.2. Phase Structure and Microscopy Characterization

The phase structure and microstructure were characterized by X-ray diffraction (Bruker D8, Germany) using Cu K*α* radiation and scanning electron microscope (Quanta FEG 250, FEI, USA) equipped with an energy dispersive spectrometer (EDS), respectively.

### 5.3. Thermoelectrical Properties Measurement

The Seebeck coefficient and electrical conductivity were measured by a ZEM-3 apparatus (UlvacRiko, Inc., Japan) under a helium atmosphere from 300 K to 853 K. The thermal conductivity was calculated from *κ* = *λρC*_*p*_, where *λ* is thermal diffusivity measured by the laser flash method (NETZSCH LFA-457, Germany), *ρ* is the sample density determined by the Achimedes method, and *C*_*p*_ is the heat capacity calculated by the Dulong-Petit law. The Hall coefficient (*R*_*H*_) at room temperature was measured using a Quantum Design physical properties measurement system (Quantum Design PPMS-9, USA) in magnet fields from -5 T to 5 T. Hall carrier coefficient (*n*_*H*_) and mobility (*μ*_*H*_) were calculated from *n*_*H*_ = 1/(*eR*_*H*_) and *μ*_*H*_ = *σR*_*H*_, where *e* is the electron charge.

### 5.4. Magnetic Properties Measurement

Magnetization measurements were performed by a superconducting quantum interference device magnetometer from Quantum Design, MPMS (magnetic property measurement system) under magnetic field of 200 Oe from 0 to 300 K, and the rate of heating and cooling is 1 K/min. The Seebeck coefficient of low temperature (from 50 K to 275 K) was using a physical property measurement system (Quantum Design PPMS-9, USA) from 50 to 300 K and the rate of heating and cooling is 0.5 K/min.

## Data Availability

All data needed to evaluate the conclusions in the paper are presented in the paper and supplementary materials. And additional data are available from the corresponding authors upon reasonable request.

## Supplementary Materials

Supplementary Materials A. Power XRD pattern and Lattice constant for Ge1-yBiyMnTe1.94Se0.06. B. SEM image of GeMnTe1.94Se0.06 and Ge0.94Bi0.06MnTe1.94Se0.06 after annealing. C. The electrical transport properties of GeMnTe2-*x*Se*x* (*x* = 0, 0.02, 0.04, 0.06, 0.08) and Ge1-*y*Bi*y*MnTe1.94Se0.06 (*y* = 0, 0.04, 0.06, 0.08). D. The magnetic properties of GeMnTe2-*x*Se*x* (*x* = 0, 0.06).
