# Supplementary material for: Anomalous Thermopower and High ZT in GeMnTe2 Driven by Spin's Thermodynamic Entropy
Source: Research (Wash D C). 2021 Mar 11;2021:1949070. doi: 10.34133/2021/1949070 (PMC7980773; doi:10.34133/2021/1949070)
Supplement: Supplementary Materials — A. Power XRD pattern and Lattice constant for Ge1-yBiyMnTe1.94Se0.06. B. SEM image of GeMnTe1.94Se0.06 and Ge0.94Bi0.06MnTe1.94Se0.06 after annealing. C. The electrical transport properties of GeMnTe2-xSex (x = 0, 0.02, 0.04, 0.06, 0.08) and Ge1-yBiyMnTe1.94Se0.06 (y = 0, 0.04, 0.06, 0.08). D. The magnetic properties of GeMnTe2-xSex (x = 0, 0.06). [file 1949070.f1.pdf]

Supplemental Information:

Anomalous thermopower and high *ZT* in $GeMnTe_2$ driven by spin's thermodynamic entropy

Sichen Duan,[1,3] Yinong Yin,[1,2] Guo-Qiang Liu,[1,2,*] Na Man,[1,2] Jianfeng Cai,[1] Xiaojian Tan,[1,2] Kai Guo,[3] Xinxin Yang,[3] and Jun Jiang[1,2,**]

[1]*Ningbo Institute of Materials Technology and Engineering, Chinese Academy of Science, Ningbo 315201, China.*

[2]*Center of Materials Science and Optoelectronics Engineering University of Chinese Academy of Sciences, Beijing 100049, China.*

[3]*School of Materials Science and Engineering, Shanghai University, Shanghai 200444, China.*

*Corresponding authors: liugq@nimte.ac.cn

**Corresponding authors: jjun@nimte.ac.cn

A. Power XRD pattern and Lattice constant for $Ge_{1-y}Bi_yMnTe_{1.94}Se_{0.06}$

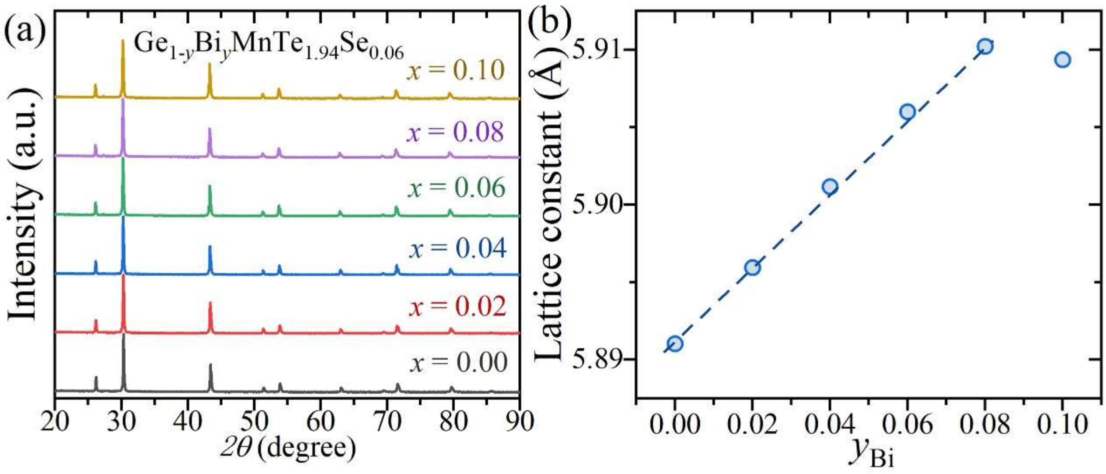

Figure S1. Power XRD patterns and lattice constant for $Ge_{1-y}Bi_yMnTe_{1.94}Se_{0.06}$. (a) Power XRD patterns, (b) lattice constant as a function of Bi doping.

B.SEM image of $GeMnTe_{1.94}Se_{0.06}$ and $Ge_{0.94}Bi_{0.06}MnTe_{1.94}Se_{0.06}$ after annealing.

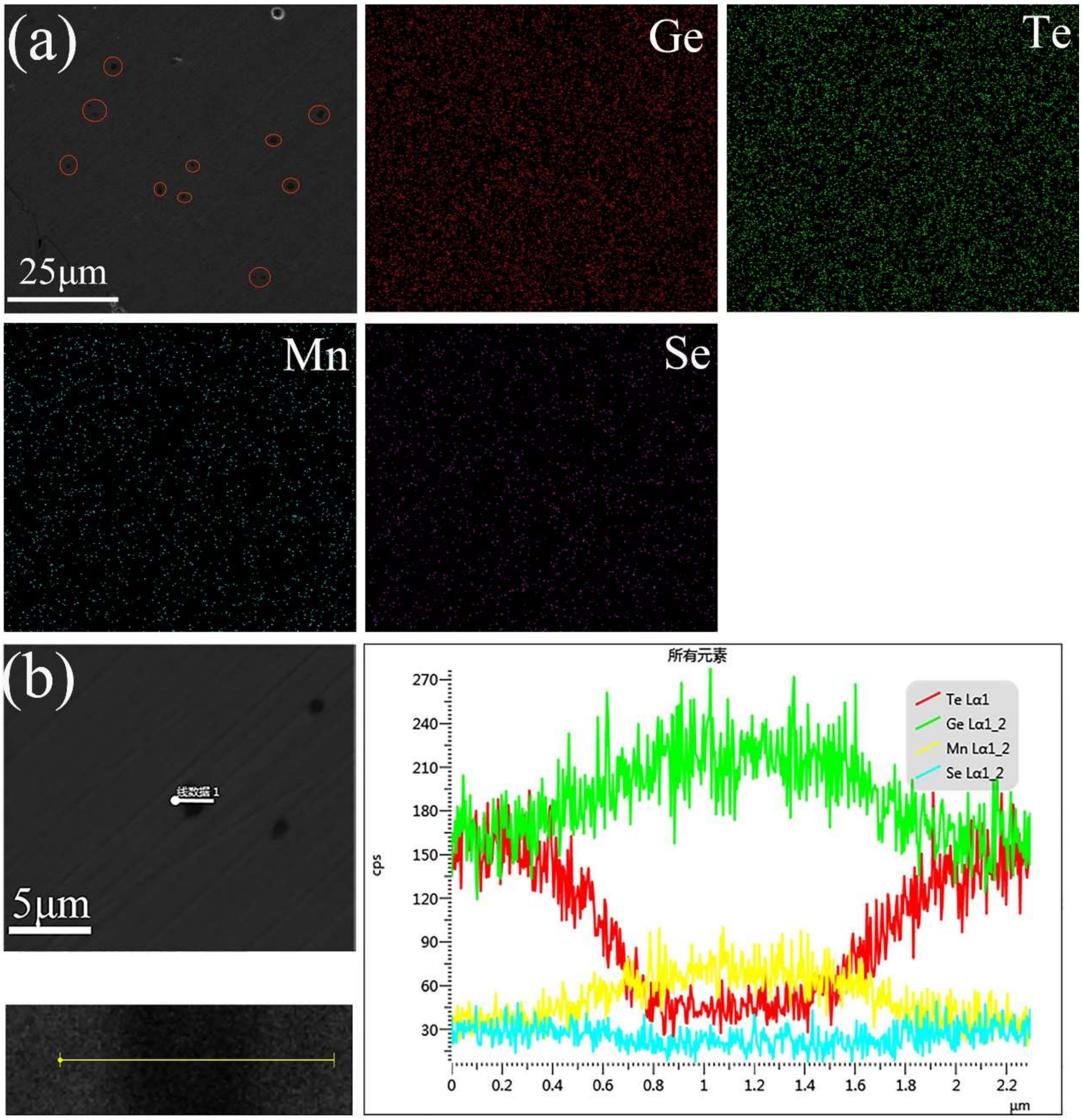

Figure S2. Chemical identification of the $GeMnTe_{1.94}Se_{0.06}$ sample. (a) SEM and elemental EDS mapping, the red circle marked is the second phase. (b) Enlarge image and EDS line profile of the second phase.

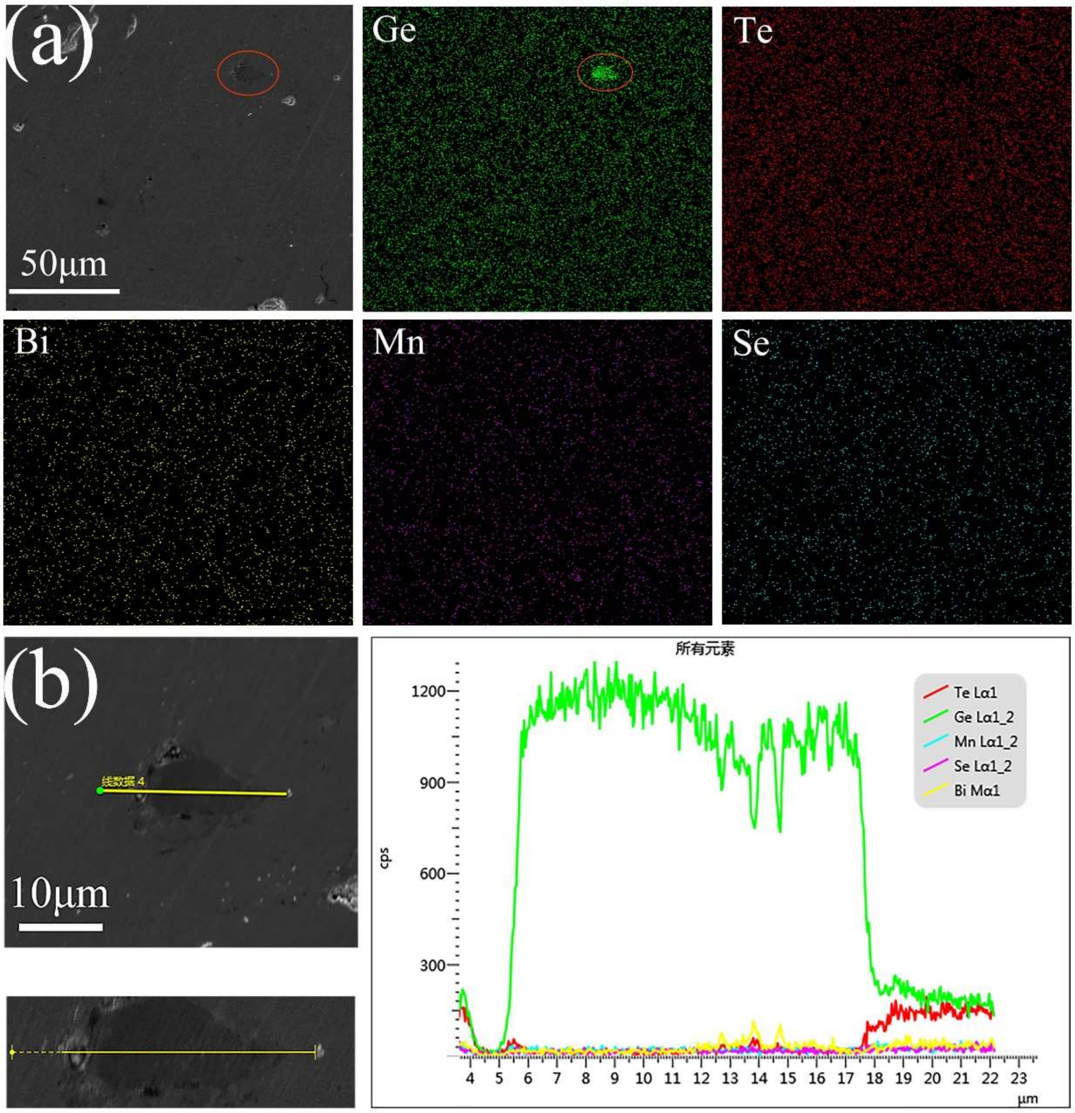

Figure S3. Chemical identification of the $Ge_{0.94}Bi_{0.06}MnTe_{1.94}Se_{0.06}$ sample. (a) SEM and elemental EDS mapping, the red circle marked is the second phase. (b) Enlarge image and EDS line profile of the second phase.

C. The electrical transport properties of $GeMnTe_{2-x}Se_x$ (x = 0, 0.02, 0.04, 0.06, 0.08) and $Ge_{1-y}Bi_yMnTe_{1.94}Se_{0.06}$ (y = 0, 0.04, 0.06, 0.08)

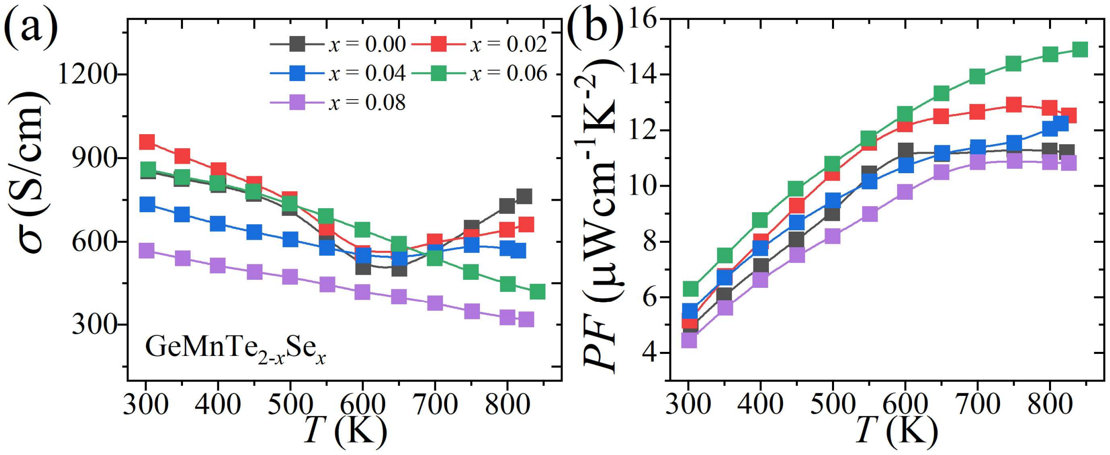

Figure S4. The electric properties of $GeMnTe_{2-x}Se_x$. (a) The temperature-dependent electrical conductivities and power factors of $GeMnTe_{2-x}Se_x$ (x = 0, 0.02, 0.04, 0.06, 0.08).

D. The magnetic properties of $GeMnTe_{2-x}Se_x$ (x = 0, 0.06)

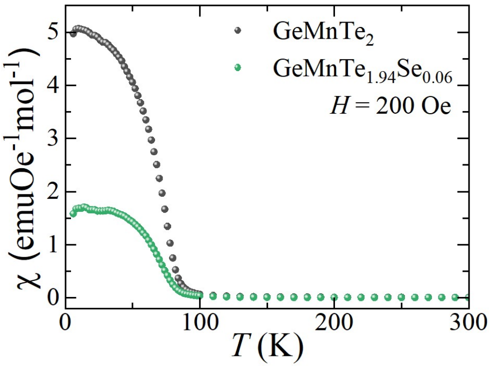

Figure S5. The temperature-dependent magnetic susceptibilities of $GeMnTe_{2-x}Se_x$ (x = 0, 0.06).
